# A Cryostat Applicable to Long-Wavelength Light-Driven Scanning Probe Microscopy

**DOI:** 10.3390/mi14020378

**Published:** 2023-02-02

**Authors:** Kui Xiang, Caihong Xie, Qiyuan Feng, Ze Wang, Guangbin Dai, Jihao Wang, Jing Zhang, Wenjie Meng, Yubin Hou, Qingyou Lu, Yalin Lu

**Affiliations:** 1 High Magnetic Field Laboratory, Hefei Institutes of Physical Science, Chinese Academy of Sciences, Hefei 230031, China; 2 University of Science and Technology of China, Hefei 230026, China; 3 Anhui Province Key Laboratory of Condensed Matter Physics at Extreme Conditions, High Magnetic Field Laboratory and High Magnetic Field Laboratory of Anhui, Hefei 230031, China; 4 Anhui Laboratory of Advanced Photon Science and Technology, University of Science and Technology of China, Hefei 230026, China; 5 Hefei National Research Center for Physical Sciences at the Microscale, University of Science and Technology of China, Hefei 230031, China

**Keywords:** cryostat, scanning probe microscopy, long-wavelength light, ANSYS, magnetic field

## Abstract

Recently, there has been growing interest in using lightwave-driven scanning probe microscopy (LD-SPM) to break through the Abbe diffraction limit of focusing, yielding insight into various energy couplings and conversion processes and revealing the internal information of matter. We describe a compact and efficient optical cryostat designed for LD-SPM testing under magnetic fields. The exceptional multilayer radiation shielding insert (MRSI) forms an excellent temperature gradient when filled with heat conducting gas, which removes the requirement to install an optical window in the liquid helium cooling shell. This not only critically avoids the vibration and thermal drift caused by solid heat conduction but also minimizes light transmission loss. The application of gate valves and bellows allows a simpler and more effective replacement of the sample and working cell in the test cavity. ANSYS software is used for steady-state thermal analysis of the MRSI to obtain the temperature distribution and heat transfer rate, and the necessity of the flexible copper shielding strips is illustrated by the simulations. The topography and magnetic domain images of 45 nm-thick La_0.67_Ca_0.33_MnO_3_ thin films on NdGaO_3_(001) substrates under a magnetic field were obtained by a self-made lightwave-driven magnetic force microscope in this cryostat. The resolution and noise spectra during imaging reveal temperature stability and low vibration throughout the cryostat. The experience acquired during the development of this cryostat will help to establish cryostats of similar types for a variety of optic applications requiring the use of cryogenic temperatures.

## 1. Introduction

The complex optical constants of some key functional materials in the infrared and terahertz frequency band can be directly related to the characteristic properties of the material (such as the superconducting energy gap and inter-band transitions), a property that other frequency bands lack [1,2]. Therefore, this correlation can be used to achieve high-resolution, high-sensitivity, precise testing of the physical properties of functional materials [3]. Lightwave-driven scanning probe microscopes (LD-SPM) are an emerging electromagnetic microscope based on the above correlation, and its sharp microprobe can localize the electric field of incident radiation on the nanometer scale, well beyond the Abbe diffraction limit [4,5,6]. This device’s excellent capabilities open new avenues for exploring new effects and new mechanisms of quantum materials, especially for high-temperature superconducting materials [7,8] that have been practically applied in the fields of transmission, quantum theory, and magnetic confinement, as well as multiferroic and giant magnetoresistance materials which have great prospects in the fields of multi-state information storage and quantum theory [9,10]. However, the above-mentioned materials require an extremely cold environment, and a magnetic field is applied during LD-SPM imaging.

There are three commonly used cryogenic systems for optical spectroscopy experimentation at low temperatures: flow cryostats, closed-cycle helium refrigerators, and bath cryostats [11]. Each has certain advantages and inherent restrictions. For example, the continuous flow cryostat has a wide temperature range but requires helium transfers and consumes large amounts of liquid helium during cooling and operation [12]. Closed-cycle helium refrigerators are self-contained and easy to use, but they are prone to large temperature gradients and mechanical instability produced by a compressor and the cooling system itself, making them unsuitable for strict temperature-dependent measurements or for measuring small samples [13,14]. Bath (or immersion) cryostats provide optimum temperature and mechanical stability while providing a large-caliber cryogenic space for optical experiments without high loss of liquid helium [15,16]. Cryostats filled with liquid coolants are quite compatible with ultra-high vacuum equipment, including magnets. For these reasons, a bath cryostat is the best choice for conducting light-driven scanning microscopic imaging.

Bath cryostats with optical windows are designed differently depending on how the sample is cooled [17,18,19]. Before the light beam reaches the sample, it typically needs to pass through three isolation layers: the vacuum chamber shell at room temperature, the radiation screen cooled by liquid nitrogen, and the liquid helium cooling wall. The vacuum chamber shell inevitably needs to be provided with a window. However, since the radiation screen cooled by liquid nitrogen does not need to be sealed in a vacuum, it is enough to open a through hole on the screen corresponding to the window. Whether the wall requires a vacuum-tight optical window depends on whether the sample is cooled by a conduction gas or by thermal contact with the liquid helium wall. The former will add an optical window layer, which will bring additional light transmission loss. The latter will introduce mechanical displacement and severe vibration noise caused by boiling coolant.

From the short review above, key findings emerge: the existing general-purpose optical cryostats are very unfavorable for the construction of an LD-SPM system that requires both high-intensity light and low vibration in a cryogenic environment. To break through the above bottlenecks, we have designed, built, and tested a compact cryostat with a single optical window that can be used for infrared or terahertz experiments under a magnetic field. We have designed a unique multilayer radiation shielding insert (MRSI), so that when the heat-conducting gas is filled from the test cavity to the optical window, a good temperature gradient can still be formed, and water condensation on the optical window can be avoided. This means that the cryostat requires only an optical window in the room temperature shell while using conduction gas for cooling, which not only reduces the light transmission loss, but also avoids vibration caused by solid thermal contact. In this way, the working cell can be suspended in the cryostat by a spring, which can meet the requirements for measurements at higher vibration. The use of bellows and gate valves allows rapid replacement of samples and working cells in the test cavity, improving cryogenic experimental efficiency. This cryostat is designed for use near magnets, especially cryogen-free magnets with room temperature apertures, which is of great practical importance as such magnets have become more widely used. 

To evaluate the performance of the cryostat, we tested the loss of terahertz through a single-layer optical window, and the transmittance exceeded 70% in the frequency range of 0.1 THz to 1 THz. We simulated the thermal insulation performance of the MRSI through ANSYS, which showed the superiority of the design using a flexible copper shielding strip. Then, a 45 nm-thick La_0.67_Ca_0.33_MnO_3_ (LCMO) film on a NdGaO_3_ (001) substrate was tested with a self-made LD-SPM in the cryostat. Topographical and magnetic domain images were obtained, which verified the vibration absorption capability and temperature stability of the cryostat. This cryostat solves the problem of compatibility among light intensity, sample temperature, and magnetic field intensity when performing long-wavelength light scanning microscopic imaging research. This is of great significance for the development of high-resolution and high-sensitivity precise measurement technology at cryogenic temperatures in the infrared and terahertz frequency bands. 

## 2. Apparatus Description

### 2.1. Cryostat

**Figure 1:**
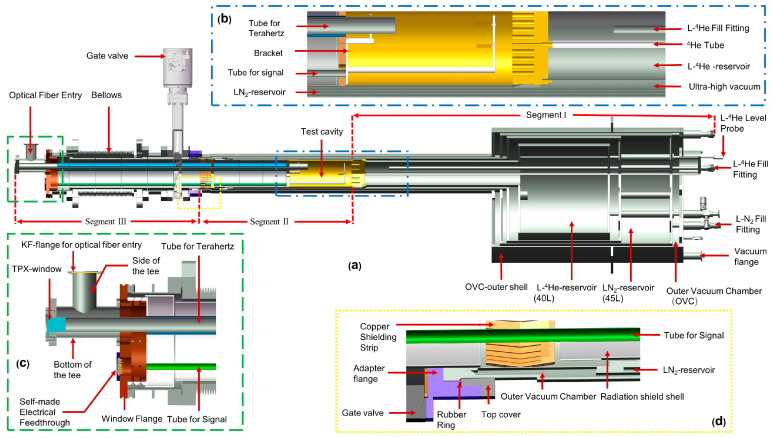
(**a**) Three-dimensional cross-section of the cryostat designed for LD-SPM. (**b**) Enlarged view of the test cavity. (**c**) Enlarged view of the window flange. (**d**) Enlarged view of the bottom of the liquid nitrogen reservoir.

Figure 1 shows the design of the cryostat we developed for LD-SPM. This cryostat is suitable for our laboratory’s cryogen-free superconducting magnet. The maximum magnetic field of the magnet is 10 T, which can meet the requirements of magnetic field modulation for most light-driven scanning microscopic imaging. The magnet has a room-temperature bore tube with an inner diameter of 150 mm. The cryostat is designed to be eccentric, which reduces its overall height when placed in the magnet. The cylindrical shell and all top plates of the cryostat were manufactured from stainless steel (SUS304). It should be emphasized that SUS304 will exhibit magnetism due to deformation-induced martensitic transformation during processing, which will generate an excessive load during the excitation process of the magnet. We ran a higher-load high-temperature furnace made of SUS304 in this magnet, and we have learned that the magnet can be safely and smoothly excited to 10 T [20]. This testing endorses the use of this cryostat in such magnets.

Due to the difference in spatial location and function, the cryostat can be described as three segments. Segment Ⅰ is a liquid helium reservoir. We added a liquid nitrogen reservoir between the liquid helium reservoir and the room temperature shell, which can reduce the temperature of the outer surface opposite to the liquid helium wall from 300 K to 77 K, reducing the radiation heat flow to the original from 1/150 to 1/200. The interlayer between the chambers is pumped into a high vacuum through the flange on the outer shell. The high vacuum of 10^−6^ torr inside the outer shell practically eliminates heat conduction by residual gas. The above-mentioned design has a series of advantages, such as small pre-cooling capacity, short stabilization time, and an excellent heat insulation effect.

Segment Ⅱ is the test cavity. The test cavity space has a diameter of 115 mm and a height of 230 mm. As shown in Figure 1b, a liquid nitrogen layer is also provided outside the test cavity, which is a downward extension of the liquid nitrogen reservoir in Segment Ⅰ. The outer shell of the test cavity is wrapped with thirty layers of insulation to further reduce the heat radiation from the vacuum chamber. The top of the test cavity is the liquid helium reservoir. A helium tube is inserted into the test cavity through the liquid helium reservoir, so the conduction gas is pre-cooled by liquid helium before entering the test cavity. In the test cavity, conduction gas is used to cool the entire working cell; this method is faster and more convenient than the more complex and vibration-transmitting cold finger device used in other equipment.

Segment Ⅲ includes gate valves, bellows, and the window flange. The upper and lower sides of the bellows are, respectively, fixed to the gate valve and window flange. The flexible structure of the bellows can not only be used to replace the working unit; it is also suitable for solid heat conduction, and its thermal insulation effect in the axial direction is better than that of a stainless-steel tube of the same length.

As shown in Figure 1c, the window flange adopts a KF (from Klein Flansche) structure in Segment III. The window is equipped with a side-outlet tee connection for the incoming terahertz signal and a self-made feedthrough for electrical signal transmission. Both the upper port side of the tee and the feedthrough are secured to the window flange with O-ring seals. The bottom of the side outlet tee is fixed with an optical window, and the side is an optical fiber entry for the transmission of infrared light. The tube for terahertz and the tube for signal in the multilayer radiation shielding insert are coaxial with the optical window and feedthrough, respectively. Infrared or terahertz and signal wires arrive at the working cell from these two channels, respectively.

Figure 1d shows the connection of joints between the bottom of the liquid nitrogen storage reservoir and the gate valve. Three parts, the OVC outer shell, the top cover, and the adapter flange, form a triangular gap, and a rubber ring (indicated by the red dot in Figure 1d) is placed in the gap. A high vacuum seal can be achieved by squeezing the rubber ring. In addition to creating a strong vacuum in the test cavity, this joint can widen the test cavity to provide more room for the working cell. To ensure the vacuum connection, the required volume of the connecting part is reduced to the greatest extent. In the case of the OVC outer shell, the outer diameter is 146 mm, and the inner diameter of the test cavity remains 115 mm in diameter, which provides the measurement device with greater operating space. This connection is specially designed for spaces that are restricted both inside and outside. Through these joints, we can insert the middle part of the cryostat into the inner bore tube of the magnet before assembling it with the bottom. The compact and detachable design of the cryostat provides its compatibility with the magnet, making it suitable for use with many forms of magnet.

### 2.2. Light Coupling

To maximize transmittance, it was necessary to adopt corresponding optical windows for light of different wavelengths in the infrared and terahertz frequency bands. As shown in Figure 1a,c, to increase the transmittance of terahertz frequencies, we used polymethylpentene (TPX) with an outer diameter of 30 mm as the optical window. TPX possesses the property that its refractive index is relatively constant as a function of wavelength. It should be noted that the windows used for terahertz optical experiments can be replaced by materials with transmission bands corresponding to the wavelength range of interest, such as quartz glass, with its excellent spectral characteristics and compatibility with visible light and infrared light, or diamond, with wide its transparency in the electromagnetic spectrum. After passing through the optical window, the light reaches the test cavity through the tube for light with a polished inner surface and gold plating. This tube passes through a stainless-steel tube with a diameter of 35 mm in the multilayer radiation shielding insert eccentrically. For light in the near-infrared frequency band, we provide the optical fiber transmission method. While providing light, the optical fiber can avoid spectral decomposition before the light reaches the sample, enabling high-resolution optical spectrum analysis, and the heat generated by the external light source is largely suppressed. The optical fiber is arranged in the gap between the stainless-steel tube and the terahertz tube.

### 2.3. Multilayer Radiation Shielding Insert

**Figure 2:**
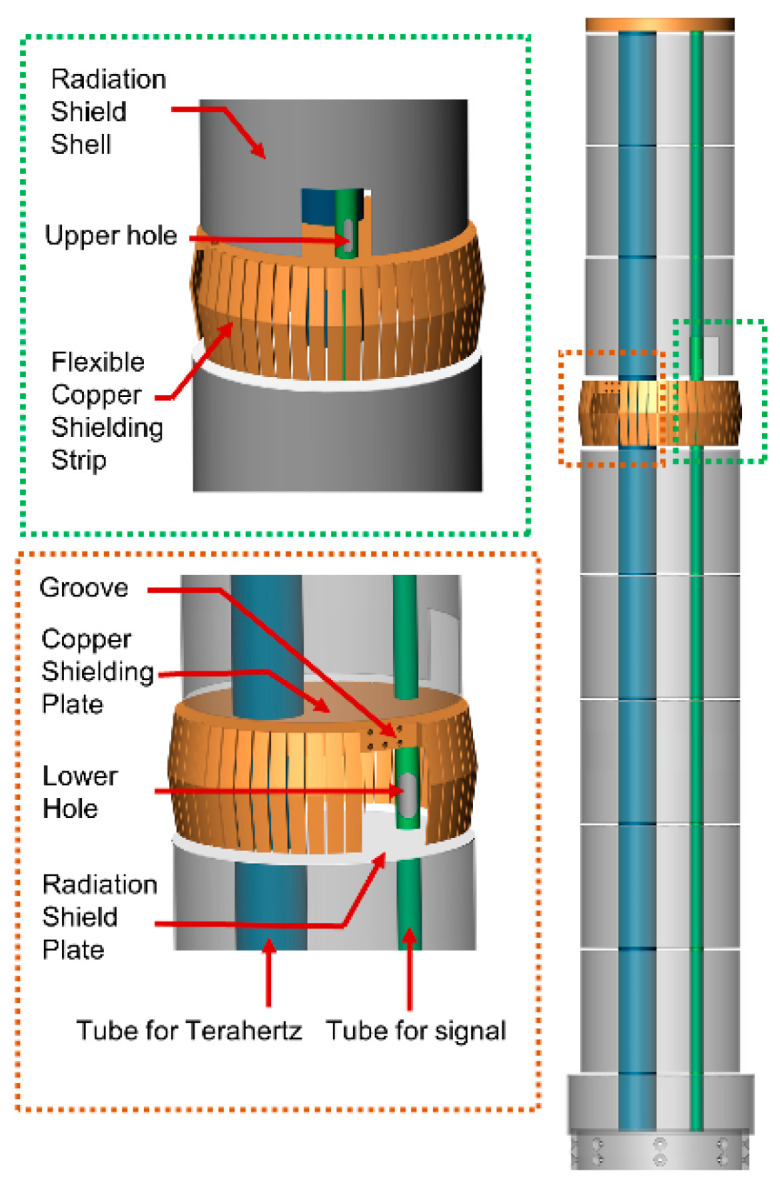
Schematic diagram of the multilayer radiation shielding insert.

As shown in Figure 2, we minimize the heat leakage caused by the exchange of gas and radiation by our design of a unique multilayer radiation shielding insert (MRSI). A circular polished plate with a thickness of 0.5 mm is used as a radiation shield sheet, and a polished stainless-steel tube with a thickness of 1 mm is used as a radiation shield shell. Both have the same outer diameter and are coaxially welded together to form a shield. Copper shielding is placed between these shields; this shield is composed of a copper shielding plate and a flexible copper shielding strip. Both are made of oxygen-free high-conductivity (OFHC) copper, which gives the structure good thermal conductivity and elasticity. The shield plate is axially supported by two stainless steel support tubes connected between the window flange and the test chamber. Depending on the inner diameter of the test cavity and the bellows, the radiation shield sheet at different positions has different outer diameters, but there must be a minimum clearance of 3 mm between the MRSI and the inner wall of the test cavity. Thick, oxygen-free, high-conductivity copper disks were used to construct the top plates to ensure a constant and homogeneous temperature.

As shown in Figure 1a,d, the MRSI is arranged coaxially with the bellows and the test cavity. The outer diameter of the copper shielding strip is slightly smaller than the inner diameter of the inner wall of the liquid nitrogen; therefore, elastic deformation will occur when the multilayer radiation shielding insert is pushed into the cryostat. At this time, the elastic copper shielding strip will be tightly pressed against the inner wall, and the liquid nitrogen will cool it through solid heat conduction. The entire MRSI will form a thermal shield at the temperature of liquid nitrogen at the copper shielding strip.

The tube for the signal also has holes on both sides of the copper shielding plate. There is a rectangular groove with threaded holes on the copper shielding side. The signal transmission wire passes through the groove from the upper hole, and then returns to the tube from the lower hole. In the rectangular groove, the wire is tightly pressed into the groove with a screw bolt. In this way, the copper shielding plate can cool the wire, further reducing heat loss through the solid conduction of the signal transmission wire.

### 2.4. Operation

The operation of this cryostat features the rapid transfer of samples or working cells from room-temperature atmospheric space to the test cavity of the cryostat. The working cell can be suspended on the bracket at the top of the MRSI through springs, but it can also be rigidly fixed directly on the heat exchange copper plate on top of the MRSI, depending on the requirements of the working cell for shock absorption and sound insulation. Then, the MRSI is fixed coaxially with the bellows through the window flange, as shown in Figure 1a,c. Then, the bellows evacuate the inner chamber to form a high-vacuum environment, and it is cleaned with high-purity helium. After repeating the cleaning operation several times, the same air pressure is maintained in the chamber as in the test cavity. Then, we open the gate valve, compress the bellows straight upwards so the MRSI carries the working cell into the test cavity, and fix the bellows after the working cell reaches the center of the magnet.

To remove the working cell from the test cavity, we stretch the bellows straight downwards to allow the working cell to fully enter the bellows, then close the gate valve. We then infuse clean ambient-temperature helium into the bellows’ inner chamber until it reaches atmospheric pressure, and after the temperature of the measuring unit has risen to room temperature, the MRSI and the working cell are pulled out of the inner chamber of the bellows. Many experimental operations have proven that the whole process is convenient and reliable, the working cell replacement process is secure and speedy, and the experimental efficiency at liquid helium temperature is enhanced, which is appropriate for measurements using an LD-SPM. Future applications of this modality of operation include extended terahertz low-temperature spectral characterization and time-resolved studies using THz-STM.

## 3. Thermal Ansys and Measurement

### 3.1. Steady-State Thermal Analysis

Segment III and the MRSI are the parts with the largest heat leakage in the whole cryostat, and this leakage is attributable to the lack of protection from the liquid nitrogen interlayer. Steady-state thermal analysis is used for evaluating the thermal equilibrium of a system or object with thermal loads that do not vary with time [21,22,23]. The steady-state thermal analysis of the MRSI placed in Segment III was carried out using the finite element software ANSYS (2019 R3).

**Figure 3:**
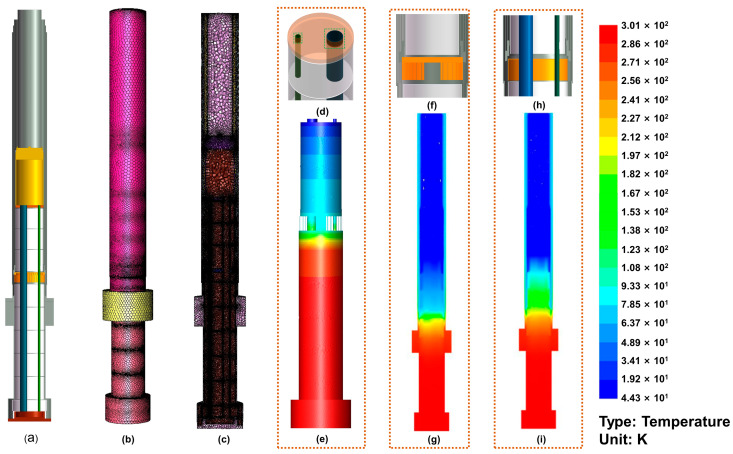
(**a**) Cross-sectional view of the simplified three-dimensional structure. (**b**,**c**) Meshing of the model in (**a**) in ANSYS. (**d**) The top copper plate section of the MRSI. (**e**) Temperature distribution of the MRSI. (**f**) Cross-sectional view of the simplified model of flexible copper shielding strip. (**g**) Temperature distribution for the model of image (**f**). (**h**) Cross-sectional view of the simplified model after replacing the flexible copper shield strip with a conventional copper shield. The conventional copper shield is not in contact with the liquid N_2_. (**i**) Temperature distribution for the model of image (**h**).

As shown in Figure 3, considering the complexity of the actual overall structure and to obtain a better mesh, we approximated the local structure without affecting the steady-state thermal analysis. We changed the copper shielding strip from a cage structure to a structure similar to other radiation shielding sheets and radiation shielding shells, and we also changed the bellows and gate valves to a stainless-steel pipe structure. Perfect contact was established between the liquid nitrogen interlayer and the flexible copper radiation shielding strip. The pressure of helium in the test cavity was 10^−3^ torr. These modifications will not cause a large error between the simulation results and the actual situation. To distinguish them in Figure 3a, the copper parts are represented in yellow in the three-dimensional structure, the stainless-steel parts are represented in gray, and the window flange is represented in red. In the simulation process, the thermal conductivity of copper was set to 387.6 W/(m·K), the specific heat capacity was 381 J/(kg·K), and the density was 8978 kg/m^3^. The specific heat capacity of stainless steel was 502.48 J/(kg·K), the density was 8030 kg/m^3^, and the thermal conductivity data at different temperatures were taken from the reference website. The convective heat transfer coefficient of helium was set to 5 w/(m^2^·K).

As shown in Figure 3b,c, the model uses a polyhedral mesh. The model is very complex; therefore, an unstructured grid is suitable. Comparing the tetrahedral unstructured grid and the polyhedral unstructured grid comprehensively, the polyhedral grid has fewer grids and higher calculation efficiency on the basis of ensuring the solution accuracy. In the meshing of this model, the number of nodes used was 20,589,994, and the number of meshes was 5,938,742.

Figure 3e shows the temperature distribution of the simulated MRSI. Our MRSI contains a crucial new design element, as shown in Figure 3f, which is the thermal shielding layer at liquid nitrogen temperature, formed by the copper radiation shielding strips in close contact with the liquid nitrogen shell. The simulated temperature distribution of the model with this design is shown in Figure 3g. To verify the importance of this design, we also simulated a model in which the copper radiation shielding plate and the flexible copper shield strip are not in contact with the liquid nitrogen shell, as shown in Figure 3h. Under the condition that other boundary conditions remain untouched, Figure 3i shows the temperature distribution of this new model after simulation. We also calculated the heat conduction at specific locations for both models. This location of the two cylindrical faces is shown in the green box in Figure 3d. This is the contact part between the top copper heat exchange plate and the two stainless steel tubes, which is the most critical position for solid heat conduction of the MRSI. After simulation, the total heat transfer rates of the models shown in Figure 3f,h at this position were 0.106 W and 0.401 W, respectively. From the comparison of temperature gradient and the heat transfer rate, it can be found that when the exchange gas fills the entire radiation shield insert, the structure using liquid nitrogen to shield the middle layer can form a better temperature gradient with less heat leakage. Therefore, the liquid nitrogen shielding intermediate layer formed by the close contact between the copper radiation shielding strip and the liquid nitrogen shell is indispensable.

### 3.2. Measurement

**Figure 4:**
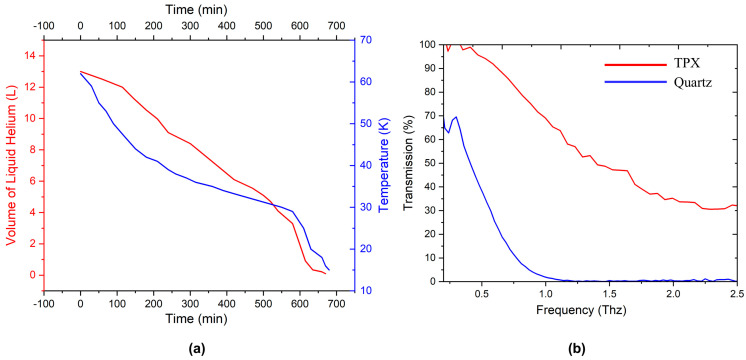
(**a**) Time dependence of temperature and liquid helium volume during cryostat cooling. (**b**) Transmittance of the terahertz frequency band through the optical windows (30 mm thick) made of different materials.

In addition to performing a steady-state thermal analysis of Segment III and the MRSI of the cryostat, we tested the time dependence of the liquid helium volume and temperature during the cryostat cooling with liquid helium. The result is shown in Figure 4a. Before cooling with liquid helium, we first pre-cooled the liquid helium reservoir with liquid nitrogen. We used a calibrated Cernox (from Lake Shore) to measure the temperature inside the test chamber. The thermometer was suspended on the bracket in the test cavity. During the test, we filled the test chamber with helium for heat exchange (the pressure in the chamber was about 10^−3^ Torr), which allows one to change the efficiency of the thermal bridge between liquid helium and the sample. As shown in Figure 4a, at 580 min, we increased the pressure of the heat conducting gas to 10^−2^ Torr, and the cooling speed and the evaporation of liquid helium were significantly accelerated. However, the pressure of the gas is not directly proportional to the cooling efficiency of the sample; it is also related to the leakage heat formed by the heat-conducting gas. The volumes of the liquid helium chamber and the liquid nitrogen vessel are 40 L and 45 L, respectively. After filling with liquid helium and liquid nitrogen, the daily loss of liquid helium and liquid nitrogen is about 12 L and 15 L, respectively. Thanks to the excellent thermal shielding performance of the MRSI, the TPX window will not have the problem of water vapor condensation during the cooling and operation of the cryostat, even when the pressure of the heat conducting gas in the test cavity reaches 10 torr.

Figure 4b shows the transmittance when the terahertz frequency band passes through the optical window. We conducted a transmittance test at normal temperature and under an atmospheric environment. Using the same thickness of quartz and TPX for comparison, the loss of TPX to terahertz was significantly smaller. At about 1 THz, the transmittance of TPX reaches 70%, while that of quartz was less than 5%. Additionally, the frequency band that TPX can pass through was wider, from 0.1 THz to 2.5 THz.

**Figure 5:**
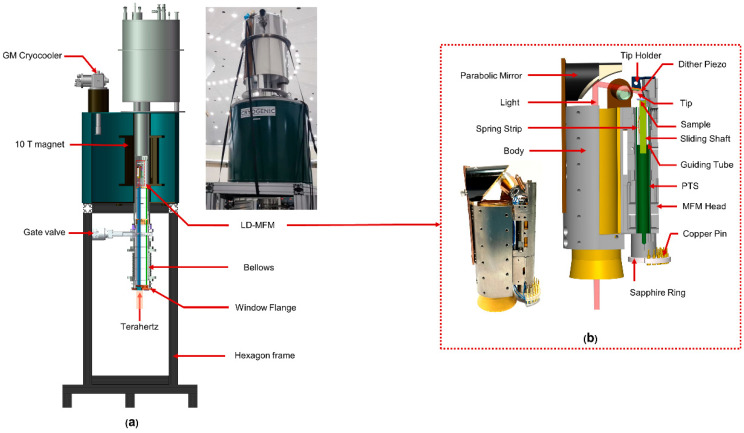
(**a**) Photo (upper right) and three-dimensional cross-sectional view (left) of the LD-SPM system in a 10 T cryogen-free superconducting magnet. (**b**) Photo (lower left) and three-dimensional model (right) of the LD-MFM.

The tip of an atomic force microscope (AFM) is typically used in LD-SPM so that topographical information and spectral information can be obtained simultaneously. We experimented with our homemade lightwave-driven magnetic force microscope (LD-MFM) in this cryostat in a 10 T cryogen-free superconducting magnet. MFM is a variety of AFM in which a sharp magnetized tip scans a magnetic sample. The tip–sample magnetic interactions are detected and used to reconstruct the magnetic structure of the sample surface. The whole device is shown in Figure 5a.

The LD-MFM is shown in Figure 5b, including the head part and the optical part. At the core of the head is a coarse-stepping motor that brings the tip closer to the sample with nanometer-scale precision. This part uses our laboratory’s self-designed inertial motor spider drive suitable for small spaces and extreme conditions; this motor includes a square rod made of high-purity tantalum serving as the sliding shaft, a guiding tube made of zirconia, a piezoelectric tube scanner (PTS), and a bent spring piece made of beryllium bronze [24,25]. We used a PTS tube with one internal electrode and four external electrodes with an outer diameter of 7.1 mm and a wall thickness of 0.55 mm (EBL 3# from EBL Products, Inc, East Hartford. This type of PTS has a large deformation under the same voltage and is more suitable for coarse step approach motors and scanning heads. The length of the PTS is 82 mm, which is much longer than that used in a traditional MFM, increasing the scanning range and making it more suitable for optical microscopic imaging.

We use a commercially available self-sensing piezoresistive cantilever as the probe (PRSA-L300-F50-STD from SCL-Sensor Tech Fabrication GmbH,, Austria). The length of the microcantilever is 305 um, the width is 110 um, the resonance frequency is 30–65 kHz, the tip radius is less than 15 nm, and the sensitivity is less than 2 μV/nm. The cantilever can directly image the sample’s topography, but to obtain the magnetic structure, a magnetic film must be deposited on the tip of the cantilever by electron beam evaporation. To do so, we used electron beam evaporation equipment (ZZS-630 from the Xing-nan technology company, China). The tip is coated with a 5 nm-thick Ti film first as the buffer layer, followed by a 50 nm-thick Co film. In order to protect the tip and increase the terahertz effect at the tip, we also wrapped 5 nm Au on the outermost layer. The probe we used is a typical cantilever beam with a conical tip, and in order to obtain a more accurate image of magnetic domains, we only coated the magnetic film on one side of the conical tip [26]. The tip is then magnetized perpendicular to the cantilever with a permanent magnet before loading into the tip holder. The cantilever is driven by a small piece of piezoelectric that is cut to the same size as the probe floor. The piezoelectric sheet is inclined at about 15 degrees relative to the sample surface to make one side (coated with the magnetic film) of the tip perpendicular to the sample surface. The signal adapter (connector) on the top of the MFM is a sheet-shaped sapphire ring with a circle of 1.5 mm holes, and the small pins of the adapter are fixed in the holes.

The optical part consists of a copper tube and a parabolic mirror. The inner surface of the copper tube is polished and gold-plated, and the parabolic mirror is fixed on top of the copper tube. The height of the mirror is adjusted so the reflected light can just reach the active area of the sample and probe. After fixing the mirror and fiber port positions at room temperature, the LD-MFM was fed into the cryostat. In this process, the optical path will not shift, since the THz part and the head part are rigidly fixed. The whole device is simple and compact in structure and very firm, with strong shock resistance; accordingly, it can be suspended from the bracket by springs.

**Figure 6:**
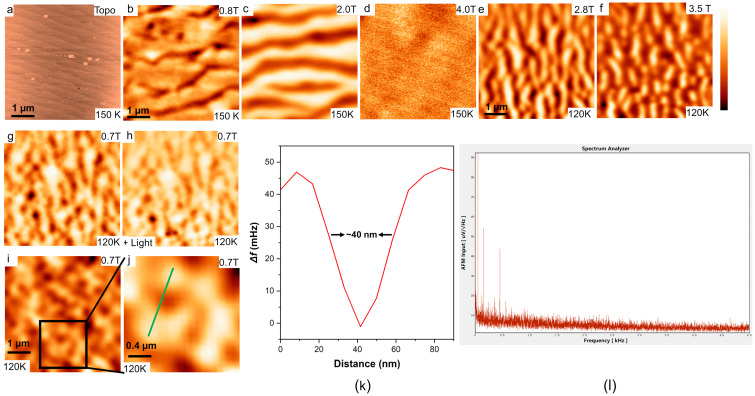
(**a**–**d**) Topography and domain images of a 45 nm-thick LCMO film at 150 K at the sample site. (**e**,**f**) Domain images of a 45 nm-thick LCMO film at 120 K at the sample site. The scale bar in images (**a**–**i**) is 1 µm and the images are 5 µm × 5 µm in size. The scale bar in image (**j**) is 0.4 µm and the image is 2 µm × 2 µm in size. The color scale for these images is 40 nm (Topo), 1.4 Hz (0.8 T), 1.6 Hz (2 T), 0.9 Hz (4 T), 3 Hz (2.8 T), 5 Hz (3.5 T), and 1.0 Hz (0.7 T). (**k**) Profile along the solid green line in image (**j**). (**l**) Noise spectrum of the output signal of the preamplifier.

We amplified the resistance change of the cantilever on the piezoresistive probe using a home-made preamplifier with a 400 times gain. The amplified signal is extracted by the built-in phase-locked loop (PLL) of the R9 controller (from RHK Technology, United States) and used for imaging [24,27].We obtained topographical and magnetic domain images of a 45 nm-thick La_0.67_Ca_0.33_MnO_3_ (LCMO) thin film on NdGaO (001) substrates using this LD-MFM in this cryostat. The sample was an epitaxial and anisotropic crystal deposited by pulsed laser deposition [27,28,29]. Stabilization of the surface temperature of the sample is provided by a resistive heater, which is controlled by the Model 336 controller (from Lake Shore Cryogenics, Inc, United States). In panels (a–h), the 5 µm × 5 µm images at the same temperature are the same area on the sample. Figure 6a–d shows the topography and magnetic pattern images when the field is swept up to μ_0_ *H* = 4.0 T at 150 K. Dark areas correspond to ferromagnetic domains (abbreviated as FMM domains), and bright areas represent antiferromagnetic charge-ordered phases (abbreviated as COI domains). The dark regions are ascribed to the FMM phase, since the attractive force between the FMM phase domains and the tip causes a negative frequency shift in the resonant frequency of the cantilever. At 4.0 T, the sample is in a fully ferromagnetic metal (FMM) state with weak contrast arising from the topography. Figure 6e–f shows the MFM images obtained at 120 K. At 2.8 T, maze-like FMM phase domains are observed. At this temperature, the FMM phase competes fiercely with the charge-ordered insulator (COI) phase, and as the magnetic field increases, the FMM phase gradually dominates over the entire surface. At 0.7 T, we irradiated the sample area with infrared and terahertz light to obtain Figure 6h. It could be found that the magnetic domains of LCMO have changed only in some small areas, the reason for this change needs to be studied with related experiments, and this article does not discuss the issue further. Figure 6k shows the profile along the solid green line in image (j). It can be calculated by measurement that its full width at half maximum is about 40 nm, which shows the high resolution of this LD-MFM. High spatial resolution is achieved thanks to our design that minimizes mechanical vibrations and results in a relatively slow drift of the sample caused by temperature changes in the upper part of the cryostat. From the noise spectrum of the output signal of the preamplifier in Figure 6l, it can be seen that the noise level and vibration level of the whole cryostat are extremely low, except for the 50 Hz originating from the power supply. The above tests verified the temperature stability, magnetic field compatibility, and excellent shock and sound insulation performance of the cryostat in microscopic measurements in the long-wavelength light frequency band.

## 4. Conclusions

We present a new optical cryostat designed for LD-SPM testing under magnetic fields, so the LD-SPM can better study the physical properties, excitation phenomena, and deep physical mechanisms of quantum materials. The cryostat uses an optical window to realize the cooling method of heat conducting gas, which not only reduces the light transmission loss, but also avoids introducing vibration noise to the working cell, making it especially suitable for research combining light and SPM. We evaluated the thermal equilibrium of the multilayer radiation shielding insert (MRSI) using steady-state thermal analysis in ANSYS, and our simulations highlight the necessity of the flexible copper shielding strips in the MRSI. We present the topography and domain images of a 45 nm-thick La_0.67_Ca_0.33_MnO_3_ (LCMO) thin film on NdGaO (001) substrates obtained using a homemade LD-MFM in this cryostat, demonstrating its temperature stability, magnetic field compatibility, and excellent shock absorption performance. The cryostat has a simple and compact structure with a novel design that satisfies the requirements for lightwave-driven scanning probe microscopy measurements under a low-temperature magnetic field. Thus, this new device has extensive practicability and will promote LD-SPM research.

## Data Availability

The data that support the findings of this study are available from the corresponding author upon reasonable request.

## References

[1] Cocker T.L., Peller D., Yu P., Repp J., Huber R. (2016). Tracking the ultrafast motion of a single molecule by femtosecond orbital imaging. Nature.

[2] Cocker T.L., Jelic V., Gupta M., Molesky S.J., Burgess J.A.J., Reyes G.D.L., Titova L.V., Tsui Y.Y., Freeman M.R., Hegmann F.A. (2013). An ultrafast terahertz scanning tunnelling microscope. Nat. Photonics.

[3] Jelic V., Iwaszczuk K., Nguyen P.H., Rathje C., Hornig G.J., Sharum H.M., Hoffman J.R., Freeman M.R., Hegmann F.A. (2017). Ultrafast terahertz control of extreme tunnel currents through single atoms on a silicon surface. Nat. Phys..

[4] Vella A., Houard J., Arnoldi L., Tang M., Boudant M., Ayoub A., Normand A., Da Costa G., Hideur A. (2021). High-resolution terahertz-driven atom probe tomography. Sci. Adv..

[5] Ammerman S.E., Jelic V., Wei Y., Breslin V.N., Hassan M., Everett N., Lee S., Sun Q., Pignedoli C.A., Ruffieux P. (2021). Lightwave-driven scanning tunnelling spectroscopy of atomically precise graphene nanoribbons. Nat. Commun..

[6] Kimura K., Morinaga Y., Imada H., Katayama I., Asakawa K., Yoshioka K., Kim Y., Takeda J. (2021). Terahertz-Field-Driven Scanning Tunneling Luminescence Spectroscopy. ACS Photonics.

[7] Zurek E., Bi T. (2019). High-temperature superconductivity in alkaline and rare earth polyhydrides at high pressure: A theoretical perspective. J. Chem. Phys..

[8] Flores-Livas J.A., Boeri L., Sanna A., Profeta G., Arita R., Eremets M. (2020). A perspective on conventional high-temperature superconductors at high pressure: Methods and materials. Phys. Rep..

[9] Wang K., Graf D., Li L., Wang L., Petrovic C. (2014). Anisotropic giant magnetoresistance in NbSb2. Sci. Rep..

[10] Sun X., Tang F., Shen X., Sun W., Zhao W., Han Y., Kan X., Cong S., Zhang L., Han Z. (2022). Anisotropic giant magnetoresistance and Fermi surface topology in the layered compound YbBi_2_. Phys. Rev. B.

[11] Meyer G.D., Ortiz T.P., Costello A.L., Brozik J.A., Kenney J.W. (2002). Simple fiber optic coupled luminescence cryostat. Rev. Sci. Instrum..

[12] Wang J., Hou Y., Geng T., Zhang J., Feng Q., Xiang K., Chen F., Luo X., Sun Y., Meng W. (2019). A variable-temperature scanning tunneling microscope operated in a continuous flow cryostat. Rev. Sci. Instrum..

[13] Naumov P.G., Lyubutin I.S., Frolov K.V., Demikhov E.I. (2010). A closed-cycle cryostat for optical and Mössbauer spectroscopy in the temperature range 4.2–300 K. Instrum. Exp. Tech..

[14] Micke P., Stark J., King S.A., Leopold T., Pfeifer T., Schmoger L., Schwarz M., Spiess L.J., Schmidt P.O., Crespo Lopez-Urrutia J.R. (2019). Closed-cycle, low-vibration 4 K cryostat for ion traps and other applications. Rev. Sci. Instrum..

[15] Gorbunov A.V., Demikhov E.I., Dorozhkin S.I., Meletov K.P., Timofeev V.B. (2009). A helium cryostat with pumping of 3He vapors for optical investigations. Instrum. Exp. Tech..

[16] Trofimov V.N., Chernikov A.N., Zaitsev-Zotov S.V., Dyuzhikov I.N., Shevlyuga V.M., Eltsov K.N. (2007). An ultrahigh-vacuum nitrogen-free helium cryostat with small heat losses. Instrum. Exp. Tech..

[17] Efimov V.B., Lokhov A.V., Mezhov-Deglin L.P. (2018). A Combined Cryostat for Neutron and Optical Investigations. Instrum. Exp. Tech..

[18] Hayashi K., Happo N., Hosokawa S. (2021). A cryostat designed for x-ray fluorescence holography experiments down to 4 K. Rev. Sci. Instrum..

[19] Rezvani S.J., Di Gioacchino D., Tofani S., D’Arco A., Ligi C., Lupi S., Gatti C., Cestelli Guidi M., Marcelli A. (2020). A cryogenic magneto-optical device for long wavelength radiation. Rev. Sci. Instrum..

[20] Wang Z., Hou Y., Feng Q., Dong H., Lu Q. (2018). High-Temperature (940 °C) furnace in 18/20 T cold bore magnet. Cryogenics.

[21] Suman N., Siddiquee A.N., Kar S. (2021). Thermal shield of the zero-boil-off cryostat for a 1.5T magnetic resonance imaging magnet. Cryogenics.

[22] Santra P., Bedakihale V., Ranganath T. (2009). Thermal structural analysis of SST-1 vacuum vessel and cryostat assembly using ANSYS. Fusion Eng. Des..

[23] Peng Y. (2011). Research of Thermal Analysis Collaboratively Using ANSYS Workbench and SolidWorks Simulation. Appl. Mech. Mater..

[24] Zhou H., Wang Z., Hou Y., Lu Q. (2014). A compact high field magnetic force microscope. Ultramicroscopy.

[25] Meng W., Guo Y., Hou Y., Lu Q. (2015). Atomic resolution scanning tunneling microscope imaging up to 27 T in a water-cooled magnet. Nano Res..

[26] Schwarz A., Wiesendanger R. (2008). Magnetic sensitive force microscopy. Nano Today.

[27] Feng Q.Y., Jin F., Zhou H.B., Wang L.F., Meng W.J., Zhang K.X., Wang J.H., Zhang J., Hou Y.B., Lu Q.Y. (2018). Induced Formation of Structural Domain Walls and Their Confinement on Phase Dynamics in Strained Manganite Thin Films. Adv. Mater..

[28] Xiang K., Hou Y., Wang J., Zhang J., Feng Q., Wang Z., Meng W., Lu Q., Lu Y. (2022). A piezoelectric rotatable magnetic force microscope system in a 10 T cryogen-free superconducting magnet. Rev. Sci. Instrum..

[29] Zhou H., Wang L., Hou Y., Huang Z., Lu Q., Wu W. (2015). Evolution and control of the phase competition morphology in a manganite film. Nat. Commun..

